# Australian Notes

**Published:** 1918-11-16

**Authors:** 


### AUSTRALIAN NOTES.

### Doctors and Lodges.

This dispute is still unsettled. As no other way out was visible, counsel for each side—the two barristers who appeared before Judge Wasley—were asked to confer. They have been conferring for over a month and have not yet reported progress. Meantime, medical institutes continue to increase and apparently to give satisfaction.

### V.D. Clinics.

The V.D. Clinics, under control of the Board of Health, opened in June, are proving successful. Six hundred and sixty new cases were reported in July, and 726 in August, with daily attendances of about 240. There are now 800 names on the books. The accommodation for men at Lonsdale Street has had to be enlarged, and instead of being open only for a few hours per week is now open from 1 to 11 P.M. every weekday. There is a department for women here as well as at the Queen Victoria Hospital, and about twenty attend daily. The necessity for a reception-house for women patients is patent. The Melbourne Hospital reports that it has 900 cases in hand at present, in- and out-patients, while at the Alfred Hospital since July 1, 1917, over 3,000 names have been registered. Dr. C. H. Johnson, medical officer in charge of the Lonsdale Street Clinic, is strongly in favour of a Lock Hospital. He was senior medical officer at Langwarrin Camp for one year and ten months, and his chief assistant, Dr. M. N. Perl, was there two years. The Board of Health reserved a day in October for a special meeting to consider steps to be taken to prevent the spreading of these diseases. The Alfred Hospital has done so well in this work that it is likely to receive a handsome State grant for the extension of buildings for their treatment.

